# From data to immunity: the role of machine learning in advancing malaria vaccine research: a scoping review

**DOI:** 10.1186/s40794-025-00271-2

**Published:** 2025-10-28

**Authors:** Shifan Khanday, Maryam Sayeed, Namra Fatma Jafri, Iqra Fatma Jafri, Raabeah Fatma Jafri, Gumana Ashraf, Sarah Safwat, Dina S. Nasr

**Affiliations:** 1 Dubai Medical College for Girls, Dubai Medical University, Dubai, 19099 United Arab Emirates; 2 Medical Parasitology Department, Faculty of Medicine, Cairo University, Cairo, Egypt

**Keywords:** Machine learning, Malaria vaccine, Antigen discovery, Immune signature, Predictive modeling

## Abstract

**Background:**

Malaria remains a significant global health burden, necessitating the development of effective vaccines. Traditional vaccine development is challenged by the complexity of the *Plasmodium* parasite and lengthy empirical processes. Machine learning (ML) offers a promising avenue to accelerate and enhance vaccine research.

**Aim:**

This review synthesizes recent advances in the application of ML to malaria vaccine research, focusing on immunological signature identification, antigen discovery, and predictive modeling of vaccine efficacy, to highlight its transformative potential.

**Methods:**

A targeted literature search was conducted for peer-reviewed articles, reviews, and systematic analyses published between 2017 and 2025. Studies directly addressing ML or AI in malaria vaccine development were included. Data extraction covered ML methodologies, data types, applications, validation strategies, challenges, and limitations. Thematic analysis categorized findings, and a quality assessment ensured methodological rigor.

**Results:**

Thematic analysis identified five key areas: (1) antigen discovery and prioritization using supervised and semi-supervised learning; (2) immune signature identification and efficacy prediction via diverse ML algorithms; (3) computational tool and framework development for data integration; (4) broad reviews of AI/ML applications; and (5) epidemiological modeling for policy support. Most studies were conducted in Europe and North America, often with collaborations in Africa.

**Conclusion:**

ML is transforming malaria vaccine research by accelerating antigen discovery, enabling precise immune profiling, and predicting vaccine efficacy. Addressing data quality, model interpretability, and validation challenges is crucial for realizing the full potential of ML in developing next-generation malaria vaccines.

**Supplementary Information:**

The online version contains supplementary material available at 10.1186/s40794-025-00271-2.

## Introduction

Malaria is a major health problem: alone in 2022 it caused more than 249 million cases and approximately 608,000 deaths [1]. *Plasmodium falciparum* (Pf) is the causal agent of almost all malaria-related deaths. Children, pregnant women and malaria-naïve subjects are at high risk of developing severe malaria, whereas adult residents of highly endemic areas develop immunity that protects from severe disease [2]. In addition, proof-of-concept studies have shown that experimental inoculation of high doses of attenuated Pf sporozoites (PfSPZ) (the mosquito-human transmission stage of the parasite) can lead to sterile protection [3]. Nevertheless, developing an effective vaccine for Pf remains a huge challenge. Pf is genetically highly divers, employs several immune evasion strategies and has a complex, multi-stage life cycle, during which more than 5,300 genes are expressed [4]. As a result, our understanding of immune responses to Pf-specific antigens that mediate naturally acquired or experimentally induced protection is incomplete.

It has been shown that up to 100% protection against controlled human malaria infection (CHMI) can be achieved by immunization of malaria-naïve adults by direct venous inoculation (DVI) of radiation-attenuated Pf sporozoites (Sanaria PfSPZ Vaccine) and by chemo-attenuated PfSPZ (Sanaria PfSPZ-CVac) [5–7]. In those studies, protection is defined as an immune state that prevents parasites from reaching the blood stage, whereas in non-protected volunteers (either non-immunized or not successfully immunized participants) parasites will invade red blood cells following an approximately 6-day-long liver stage [5]. Only the asexual blood stage of the parasite is responsible for the symptoms and complications of malaria. Pf-specific protein microarrays can be used to characterize the pattern of antibody reactivity to Pf-specific epitopes.

Machine learning (ML) is playing a transformative role in advancing malaria vaccine research by enhancing the accuracy, efficiency, and scope of vaccine development processes. In particular, machine learning methods can reduce the number of laborious tasks, estimate the effectiveness of the vaccine, and identify new vaccine candidates, which speed up the development of effective malaria interventions [8].

Malaria Screener R is another ML-based software that counts the number of Parasite-Infected Red Blood Cells in rodent models. This is important in determining the efficiency of vaccines. This minimizes human influence or bias and also allows results to be acquired at the same level of precision in a shorter time across or within different laboratories [9].

Multitask support vector machines and other ML models are applied to predict malaria vaccine efficacy, which is assessable by antibody profiles. These models can determine which antigens are considered protective aids in vaccine development, and result in better classification compared to typical procedures [10]. Utilizing such methods, ML shall help automate some time-consuming tasks, prophesy the effectiveness of the vaccines, and even define the more efficient vaccines for malaria, thus enhancing the successful development of malaria interventions [8].

Machine learning software like Malaria Screener R helps count parasites in red blood cells in the host laboratory rodent models AS, which is used to assess the efficacy of vaccines. This form of automation eliminates cognitive aspects of sample processing, as results produced are consistent and speedy across different laboratories [9]. Multitask support vector machines are utilized to identify the efficacy forms of the malaria vaccine based on antibody profiles. These sorts can recognize specific antigens that play a part in protective immunities; therefore, they are beneficial for vaccine design and possess the capability to give a better classification than the traditional one [10].

Although ML has made great progress in malaria vaccine development, issues still need to be addressed, including the requirement for massive amounts of information and the problem of transferring a single model to another site. To overcome these limitations, more research and improving the accuracy of the chosen ML models will be necessary to have a better chance of predicting and stopping malaria emergence [11]. This review systematically synthesizes recent advances in the application of machine learning (ML) to malaria vaccine research, focusing on the identification of immunological signatures, antigen discovery, and predictive modeling of vaccine efficacy.

## Methodology

### Literature search and selection criteria

**Fig. 1:**
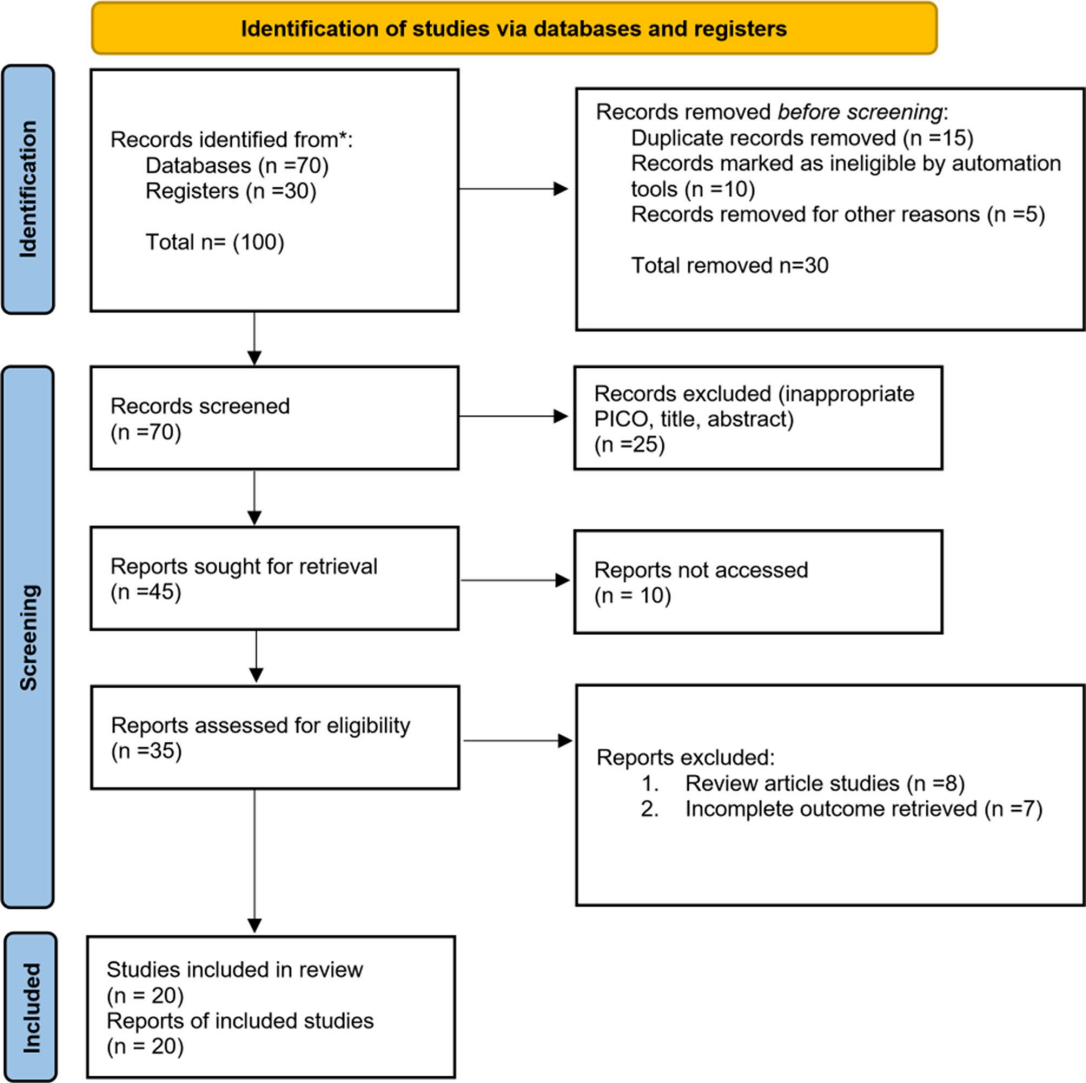
PRISMA 2020 flow diagram for new systematic reviews which included searches of databases and registers only

A targeted literature search was conducted, restricted to peer-reviewed articles, reviews, and systematic analyses published between 2017 and 2025, as listed in the reference set (Fig. 1) Inclusion criteria required that studies directly addressed the use of ML or artificial intelligence (AI) in malaria vaccine development, antigen prediction, immune profiling, or efficacy modeling. Excluded were articles focusing solely on traditional statistical approaches without a clear ML component, as well as those not specific to malaria or vaccine research.

### Data extraction and thematic analysis

Key information was extracted from each article, including:

The ML or AI methodology employed (e.g., supervised learning, unsupervised learning, positive-unlabeled learning, data integration frameworks).

The type and source of data analyzed (e.g., antibody profiles, transcriptomic data, clinical trial outcomes, pathogen genomics).

The specific application within malaria vaccine research (e.g., antigen candidate discovery, immune signature identification, efficacy prediction).

Validation strategies and performance metrics (e.g., cross-validation, ROC-AUC, accuracy, interpretability).

Reported challenges, limitations, and future directions.

### Quality assessment and synthesis

Each study was assessed for methodological rigor, including the appropriateness of ML models, robustness of validation, and clarity in reporting. Studies employing advanced or novel ML techniques (such as positive-unlabeled learning or integrative multi-omics) were given particular attention. The synthesis aimed to highlight both the technical advancements and the translational impact on vaccine research, while also identifying gaps and areas for future investigation.

## Results

**Fig. 2:**
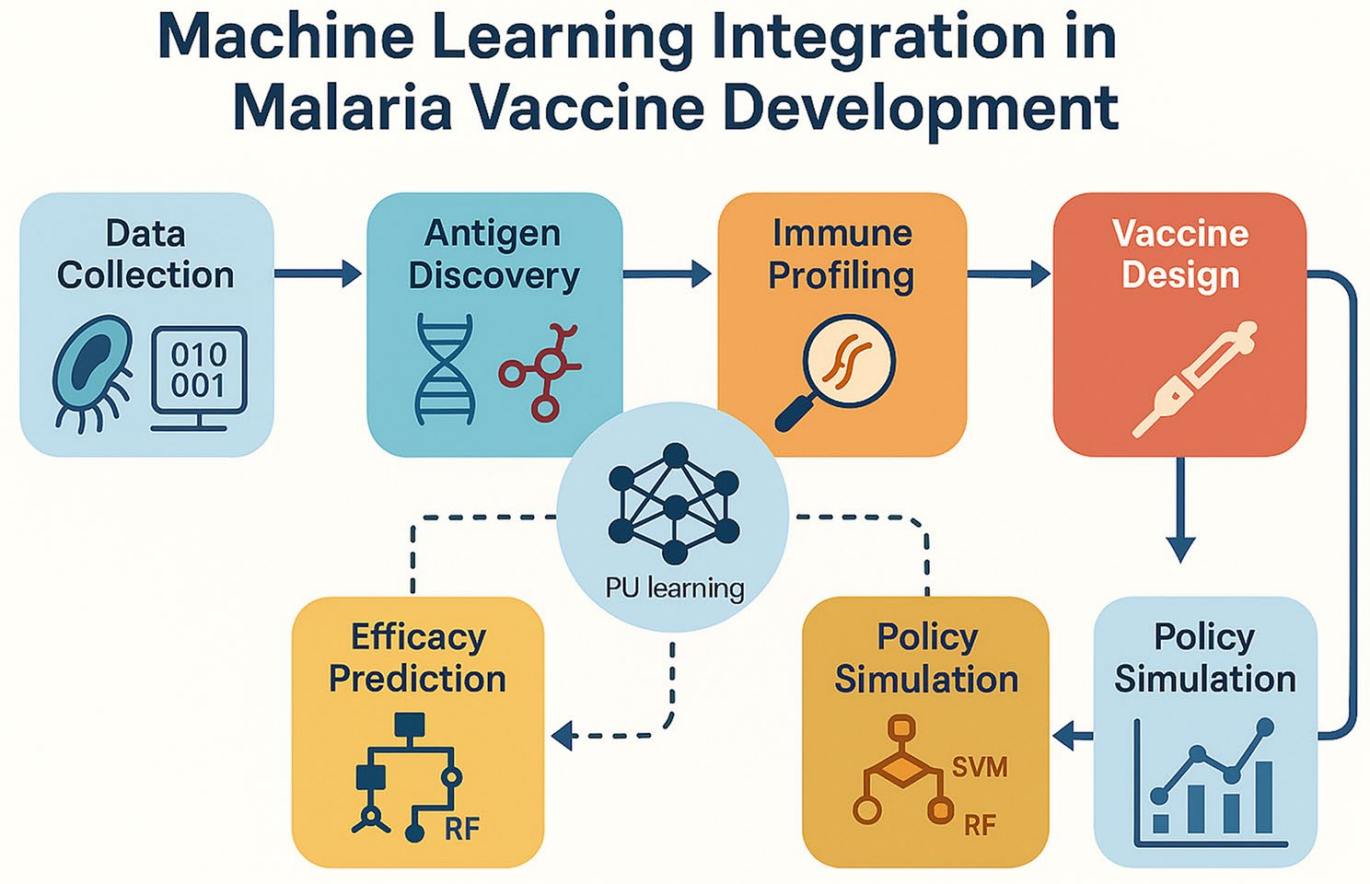
General layout for machine learning integration in Malaria Vaccine development

This section presents a thematic analysis of the studies, categorizing them according to their primary research focus within the field of malaria vaccine development and machine learning (ML). The analysis also examined the methodologies applied and the geographical context of the studies. The concept map (Fig. 2) highlights how machine learning supports antigen discovery, immune profiling, vaccine efficacy prediction, and epidemiological modeling to accelerate malaria vaccine research.

### Antigen discovery and vaccine candidate prioritization

Of the included papers, four studies were specifically dedicated to using ML to predict and rank potential malaria vaccine antigens. They were designed to compensate for the lack of studies on the life cycle of *Plasmodium* species and the lack of standard targets as dictated by vaccines [12–15].

In terms of the methodology used in these studies, machine learning approaches were mainly supervised and semi-supervised. For instance, Chou et al. (2024) applied positive-unlabeled (PU) learning, a method of semi-supervised learning that is helpful when only a subset of the positive examples (also known as antigens) are labeled, and the rest are unlabeled. This method helps to find new antigen candidates derived from the existing data if data is missing, which is common in malaria antigen studies [12]. Another work by Chou et al. expanded this research by incorporating *Plasmodium falciparum* data in predicting antigens in *Plasmodium vivax*, Fourier displaying the cross-species method to improve the model [13].

We compared the ML methods, the input data, key findings, and the limitations as summarized in Table 1.

Such studies often employ protein sequence information, immunological assays, and genomic characteristics of the samples as the independent variables that need to be predicted. Methods of validation include cross-validation and comparison with known antigen sets. The main purpose is to produce ranked lists of the potential antigens for further experimental testing, which will help to save time in the development of new vaccines.

Regionally, most of these inquiries were carried out by teams from America and Europe in association with institutions from Africa, where malaria is prevalent. This outlines a global partnership model that comprises computational know-how as well as field experience.

**Table 1:** Antigen discovery and vaccine candidate prioritization

Study (Year)	Scope/Theme	ML Method(s)	Input Data	Validation	Key Findings (as summarized in ms.)	Limitations (reported or inferred)	External Validation	References
Chou et al., 2024 (PF antigens)	Antigen discovery	Positive-Unlabeled learning (e.g., RF variant)	Protein sequence features; immunogenicity annotations	Cross-validation; comparison vs. known antigens (as described)	PU learning identifies novel P. falciparum antigen candidates	Label sparsity; generalizability	Unknown	Chou 2024 PF [13]
Chou et al., 2024 (PV antigens with PF data)	Cross-species antigen prediction	PU learning with cross-species transfer	P. vivax + P. falciparum features	Cross-validation; cross-species comparison	Adding PF data improves PV antigen prediction	Domain shift across species	Unknown	Chou 2024 PV [12, 13]
Tuju et al., 2017	Vaccine candidate discovery review	Multiple	—	—	Frameworks for next-gen malaria vaccine targets	Conceptual; limited benchmarking	N/A	Tuju 2017 [15]

### Immune signature identification and vaccine efficacy prediction

Another thematic area (5 studies) has focused on applying ML to biologically predict immunity against malaria and model vaccine effectiveness. These studies tried to disentangle the immunological signals to determine the factors associated with a certain degree of protection or construct the immunological profile of a given subject or group for a certain vaccine [10, 16–19].

**Fig. 3:**
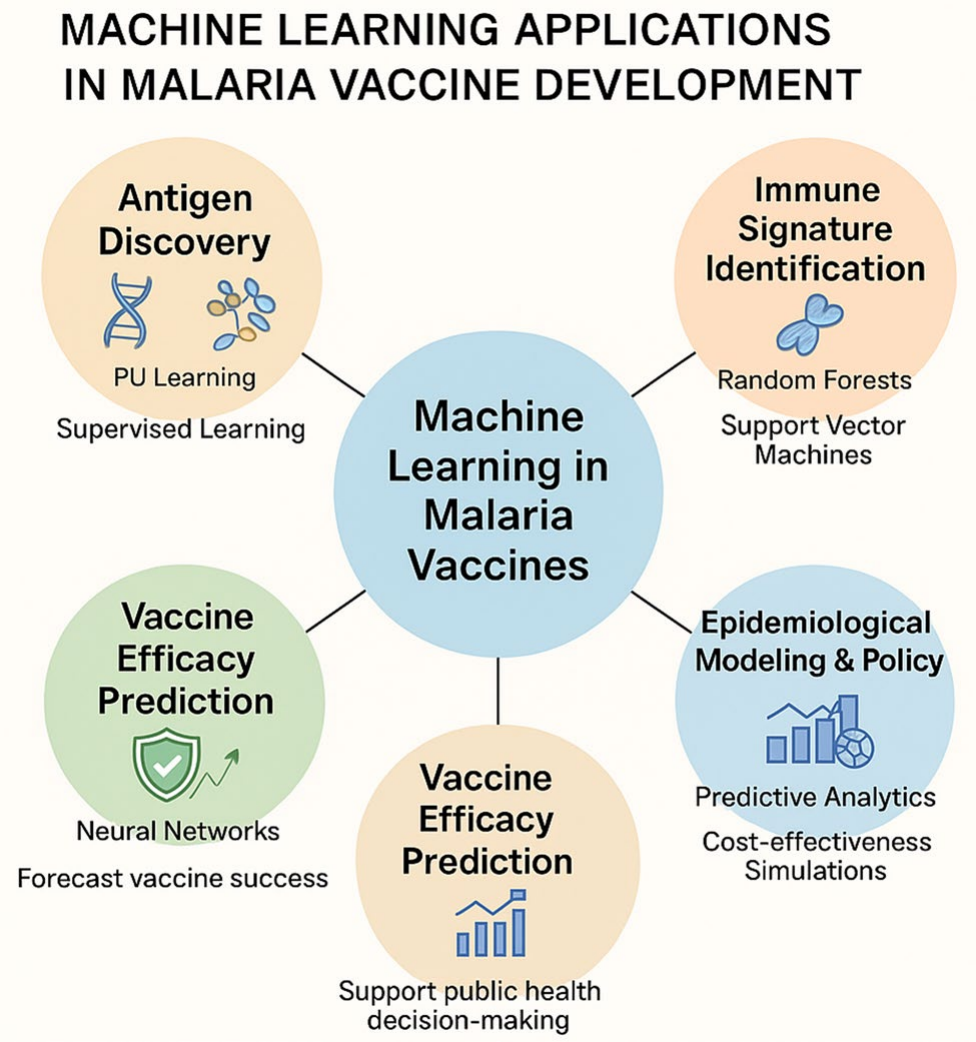
Machine learning applications in malaria vaccine development

This schematic illustration (Fig. 3) the key areas where machine learning (ML) contributes to accelerating and enhancing malaria vaccine research. ML supports **Antigen Discovery** through Positive-Unlabeled (PU) Learning and supervised approaches to identify novel vaccine targets. **Immune Signature Identification** is enabled by algorithms such as Random Forests and Support Vector Machines to uncover biomarkers of protective immunity. **Vaccine Efficacy Prediction** leverages neural networks and other models to forecast vaccine performance based on biological and immunological data. Finally, **Epidemiological Modeling and Policy Decision Support** applies ML-driven predictive analytics and simulation tools to evaluate vaccine impact, optimize deployment strategies, and inform cost-effectiveness analyses. Together, these approaches demonstrate the growing role of ML in transforming malaria vaccine development.

Most of the studies within this category employed various approaches based on Supervised learning algorithms, such as random forests, support vector machines, and neural networks. For instance, Wistuba-Hamprecht et al. used ML classifiers to predict using antibody profile data collected from clinical trials to decide on the efficacy of vaccines [10]. Similarly, Valletta and Recker 2017 employed the ML to find the classification of multivariate epithelial antibodies that correlated with clinical protection so that the ML could model high immunologic dimensions [16].

Some studies go a step further by adding the transcriptomic layer, as evidenced in van den Berg et al. (2017), where transcriptomics data from malaria challenge trials were used to develop machine learning models for RTS and S vaccine protection [17]. These methods used in integrative approaches often included dimensionality reduction as well as feature selection, especially where multiscale omics data was involved.

The statistical prediction models were further validated through internal cross-validation compared to external validation tests on other datasets, thus making the validation strong. The study aimed to identify various factors that could be used as predictors in organ transplant patients who are likely to benefit from vaccines. These studies were mostly done in Europe and North America and, in some cases, with partners in Africa, thus confirming the translational nature of this research work that combined computational modeling with immunology and human individuals.

We compared the ML methods, the input data, key findings, and the limitations as summarized in Table 2.

**Table 2:** Immune signature identification and vaccine efficacy prediction

Study (Year)	Scope/Theme	ML Method(s)	Input Data	Key Findings	Limitations	References
Wistuba-Hamprecht et al., 2024	Efficacy prediction from antibody profiles	Classifiers (e.g., RF, SVM	Multiplex antibody profiles from trials	Multi-antigen responses predict protection better than single markers	Potential overfitting; need external validation	Wistuba-Hamprecht 2024 [10]
Valletta & Recker, 2017	Immune signature discovery	Supervised learning	Multivariate antibody datasets	Identifies correlates of clinical protection	Generalizability; limited cohorts	Valletta & Recker 2017 [16]
van den Berg et al., 2017	Transcriptomic predictors of RTS, S protection	Supervised models with feature selection/dimensionality reduction	Whole-blood transcriptomes from CHMI	NF-κB / IFN-γ signaling associated with protection	Cohort size; platform effects	van den Berg 2017 [17]

### Computational tools and data integration frameworks

Three studies focused on developing computational tools and frameworks and data integration strategies for further vaccine development. These works were centered on a theoretical framework to coordinate the access and analysis of the complex and extensive data common in malaria vaccines [20–22].

Most of these undertakings employed narrative or systematic review methods to identify and model existing computational approaches and frameworks for integrating multiple omics, clinic, and epidemiological data. The focus was made here to describe bottlenecks and potential in the current computational setting and to promote AI platforms for candidate identification, immune characterization, and prediction of efficacy. These reviews also propose that transparent data repositories to be obtained through collaboration and simpler interpretation models for ML to improve trust and usability. Most of the contributions were initiated by authors based in Europe and North America, which may be because most computational biology research activities are primarily conducted by authors based in North America and Europe.

We compared the ML methods, the input data, key findings, and the limitations as summarized in Table 3.

**Table 3:** Computational Tools, data integration Frameworks, and epidemiological modeling

Study (Year)	Scope/Theme	ML Method(s)	Input Data	Key Findings	Limitations	References
Anderson et al., 2025	Computational tools & data integration (review)	Multiple	Multi-omics, clinical, epidemiological	Frameworks to integrate data and accelerate development	Interoperability; transparency	Anderson 2025 [20]
Galactionova et al., 2021	Policy modeling for RTS, S deployment	Mathematical modeling + ML components	Epidemiological and trial data	Cost-effectiveness and coverage strategy insights	Assumption sensitivity; data gaps	Galactionova 2021[23, 24]
Melchane et al., 2024	AI for infectious disease prediction	Multiple	Surveillance/real-world data	Role of ML in outbreaks & prevention	Data quality; generalization	Melchane 2024 [25]

### Systematic reviews and narrative reviews on ML applications in vaccine development

Six studies provide broad overviews of the role of AI and ML in infectious disease and vaccine development, including malaria. These reviews synthesized the state-of-the-art methodologies, highlight emerging trends, and discuss potential applications and limitations [21, 25–29].

Methodologically, these are narrative or systematic reviews that collate findings from multiple primary studies, focusing on ML algorithms such as deep learning, ensemble methods, and reinforcement learning. The aims of these reviews were to provide a comprehensive understanding of how ML can be leveraged across the vaccine development continuum and to identify gaps in current research, such as the need for better data quality and model interpretability. Geographically, these reviews originated from a diverse set of countries, including India, Nigeria, and the United States, reflecting a global interest in AI applications for malaria vaccine research.

### Epidemiological modeling and policy decision support

Two studies focus on the use of ML and computational modeling to support malaria vaccine policy decisions and epidemiological predictions. These studies apply ML techniques to large-scale epidemiological and clinical trial data to model vaccine impact and optimize deployment strategies [24, 25].

Galactionova et al. (2021) employ mathematical modeling combined with ML to simulate the effects of RTS, S vaccine implementation under different scenarios, aiming to inform policymakers on cost-effectiveness and coverage strategies [13, 24]. Melchane et al. (2024) provides a comprehensive review of AI methods for infectious disease prediction and prevention, including malaria, emphasizing the role of ML in forecasting outbreaks and guiding public health interventions [25].

The methodologies include predictive analytics, simulation modeling, and integration of real-world data streams. Validation is often performed through retrospective analyses and scenario testing. These studies are typically conducted by interdisciplinary teams based in Europe and North America, often collaborating with international health organizations.

### Geographic distribution of studies

Across the 20 studies analyzed, the majority are conducted or led by research groups in Europe (approximately 9 studies) and North America (approximately 8 studies), reflecting strong computational and immunological research infrastructures in these regions. Several studies involve collaborations with African institutions (about 5 studies), which is critical given the endemicity of malaria on the continent and the need for context-specific data.

A smaller number of studies originate from Asia (notably India) and Africa, primarily in the form of narrative reviews or systematic reviews addressing AI applications in vaccine development. This distribution highlights both the global interest in ML-driven malaria vaccine research and the ongoing need to build capacity and data resources in malaria-endemic regions.

## Discussion

### Predictive modeling in machine learning for malaria vaccine research

Malaria continues to exert a devastating toll on global health, with millions of new cases and hundreds of thousands of deaths each year, particularly in low- and middle-income countries. Despite significant advances in malaria control, the development of highly effective vaccines remains a critical yet elusive goal. Traditional vaccine discovery and development processes are currently protracted, resource-intensive, and limited by the competence of malaria parasite biology. In this context, machine learning (ML) has emerged as a transformative methodology that can study huge and complicated biological information, facilitate antigen discovery, and optimize vaccine design [30].

### Machine learning for antigen prediction: from empirical discovery to data-driven insights

**Fig. 4:**
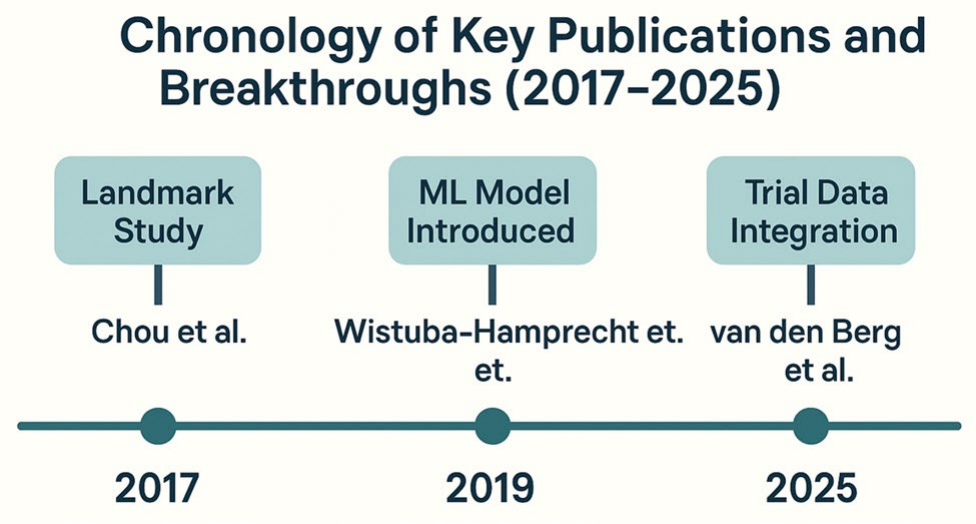
Chronology of Key Publications and Breakthrough

Specific antigens are widely thought of as the key markers that automatically define the epitome of an ideal vaccine target. In the past, this had depended mainly on trial and error coupled with an elaborate process of experimentation, which restricted the discovery process in terms of speed and scope. ML has revolutionized this, which helps to predict various vaccine candidates systematically on the basis of highly biological data. Such enhancements show the efficiency and effectiveness of ML in predicting antigens in the recent past. For instance, Chou (Fig. 4) and colleagues created a positive-unlabeled random forest (PURF) approach, which uses cross-species form for predicting antigens in P. vivax and utilizes information on its related species, P. falciparum. This integrative method enhanced the accuracy of antigen prediction; several species datasets should be used to increase the candidate number [12, 13]. Compared to most other approaches that heavily rely on antigens and their analogical similarity to known pathogens, such ML models can reveal targets that are not discernible through conventional strategies.

**Fig. 5:**
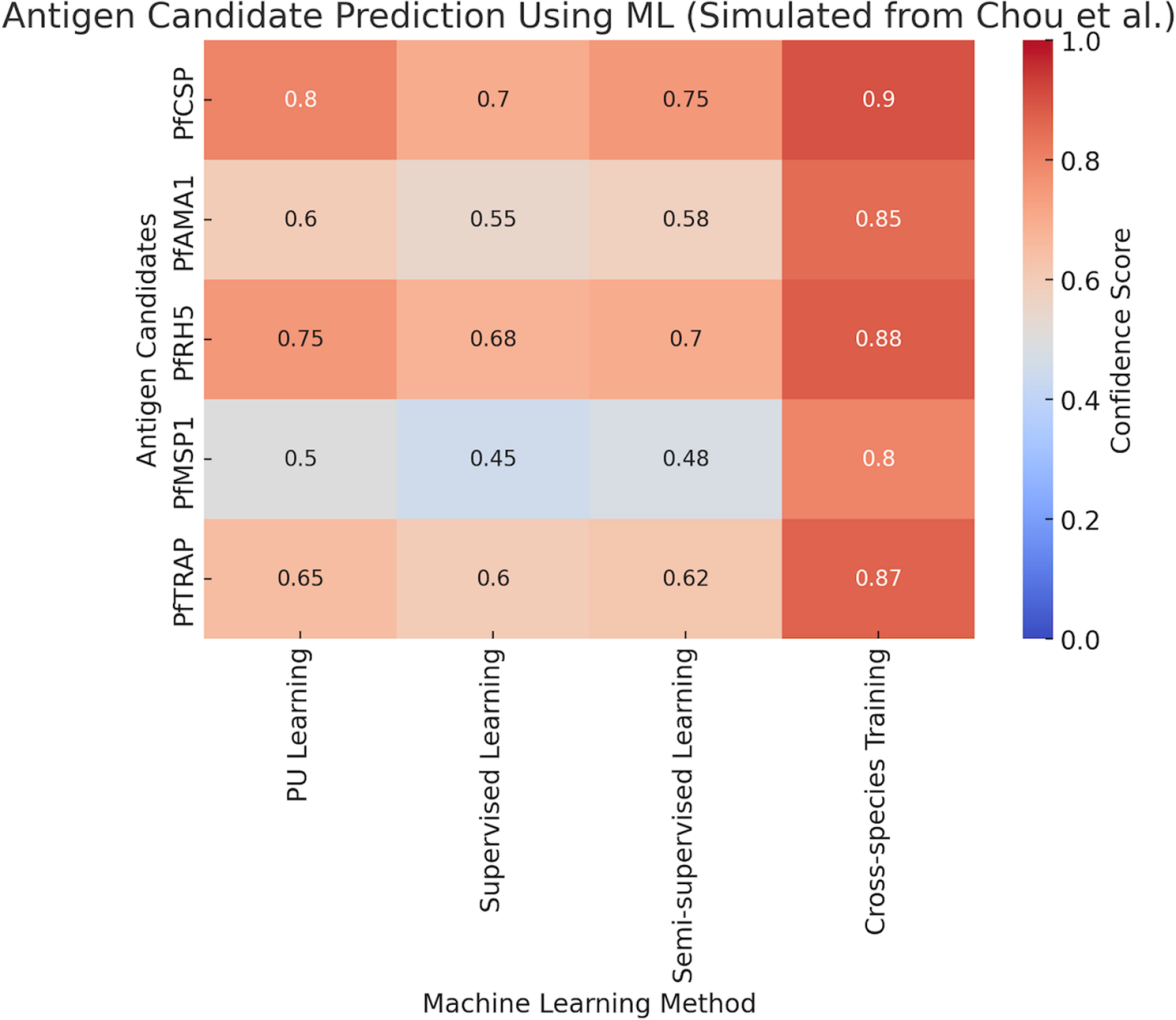
Predicted confidence scores for malaria vaccine antigens using machine learning approaches

This heatmap illustrates the simulated confidence scores for five *Plasmodium falciparum* antigen candidates (PfCSP, PfAMA1, PfRH5, PfMSP1, and PfTRAP) predicted using various machine learning (ML) approaches as shown in Fig. 5. The methods compared include Positive-Unlabeled (PU) Learning, Supervised Learning, Semi-Supervised Learning, and Cross-Species Training incorporating *Plasmodium vivax* data. Higher confidence scores (depicted in red) indicate stronger predicted suitability of an antigen as a vaccine candidate, while lower scores (depicted in blue) suggest reduced likelihood. The data shown is based on conceptual trends described by Chou et al. (2024) and does not represent actual raw experimental data. Cross-species data integration notably enhances prediction confidence for several antigens, supporting the utility of evolutionary conservation in ML-driven antigen discovery.

In addition, ML methods are not only applied to antigen prediction in the species of interest. Such cross-learning in different pathogens is done in the real sense, as exhibited in the previous studies, showing the flexibility and expansiveness of the ML frameworks in the identification of vaccines. In this regard, integrating different types of information at a proteomic level, such as immunogenicity traces, as well as the degree of conservation, will help reduce computational ML models for the selection of antigens with the highest potential for the generation of protective responses and will minimize the amount of experimental testing [12, 13].

### Predicting immune response effectiveness: decoding complex immunological signatures

Apart from identifying the antigens to be targeted and incorporated into the vaccine, a significant difficulty of malaria vaccine development is the ability to estimate the immunogenicity of the immune response generated by the candidate vaccines. In this field, ML has been helpful in investigating the vectors of high-dimensionality immunology and detecting the prognostic biomarkers of protection.

Another notable work by Wistuba-Hamprecht et al. analyzed the trends in antibody profiling from malaria vaccine trials and used ML algorithms to identify that overall multiantigen responses are more protective than single antibodies against the diseases [10]. This adds to our understanding that it is more relevant to assess the whole range and quality of markers when evaluating the immune response instead of individual ones. The immunological measurements, along with the ML models like the support vector machines or the ensemble classifiers, can bring better reliability to predicting vaccine efficacy than statistical strategies.

Next, transcriptional studies have also sought the help of ML applications. In their work, Van den Berg et al. used ML to detect the expression profile of tens of thousands of genes from clinical trials of the RTS, S malaria vaccine. They focused on the NF-κB and IFN-γ signaling pathways, which are statistically significant for vaccine efficacy [17]. These discoveries give a detailed explanation of the immune response following vaccination and the basis for future vaccines.

The characteristics of immune responses collected and analyzed by ML mean a conceptual revolution in evaluating vaccines. The model finds complex, multi-factorial patterns characterizing the response. Rather than relying on a handful of predefined correlates of protection, researchers can now interrogate the full complexity of the host response, leading to more robust and generalizable predictors of vaccine success [16].

### Accelerating antigen selection and vaccine design: integrative data analysis and reverse vaccinology

Another vital breakthrough made by ML in malaria vaccine studies is the improvement of the pace for determining antigens and designing vaccines. Besides, the general process of antigen identification is slow and requires substantial laboratory work in combination with functional tests. This process was transformed by new possibilities of using ML-based reverse vaccinology to quickly identify the desired vaccine candidates. For example, Chou et al. have proved that ML models would enable the combined analysis of the three aspects, including protein structure, function, and immunogenicity, for identifying and ranking *Plasmodium falciparum* antigens with high vaccine potency [13]. Through computational methods, large-scale proteomic and transcriptomic data can be analyzed more effectively than using a labor-intensive experimental approach, freeing resources for other promising prospects.

Furthermore, ML was observed to be flexible in that it allows the integration of different types of data, genomic, proteomic, immunologic, epidemiologic, to give a comprehensive understanding of the prioritization of antigens. This synergistic approach not only enhances the identification rate of these antigens but also increases the chances of exposing those that can stimulate an immune response and are conserved among different populations of the parasite, which are some of the hurdles in malaria vaccine development [13].

### Simulating clinical trials and optimizing vaccination strategies: the role of predictive modeling

ML has also been used to simulate clinical processes or test clinical trials, as well as optimize vaccinations. Mathematical and computational models based on ML algorithms can identify the effectiveness and possible impact of candidate vaccines and be helpful in making decisions on political and investment aspects. In a modeling study, the effectiveness and potential use of the RTS S vaccine were proven to be efficient and effective in reducing malaria transmission [23, 24]. These performances are essential in decision-making regarding personnel distribution and the interface between vaccines and other modes of malaria control.

In addition, complexities of molecular as well as dynamics have been understood and explained with the help of Machine learning, which helps dissect the immune response of vaccines. Research on transcriptional samples of the PfSPZ vaccine trials has also shown that biomarkers call for protection during clinical trials; baseline innate immunity biomarkers are particularly interested in understanding host factors that determine the vaccine’s effectiveness [18]. Besides, such insights help develop new vaccines, use ML to analyze dosing regimens, and select individuals who would benefit from a vaccine the most.

### Impact on research outcomes: toward more effective and rapidly developed vaccines

The integration of ML into malaria vaccine research has yielded significant improvements in research outcomes, spanning vaccine efficacy, development timelines, and mechanistic understanding.

### Improved vaccine efficacy

By enabling the identification of antigens and immune signatures associated with robust protection, ML has directly contributed to the design of more effective vaccines. The ability to predict which antigen combinations elicit sterile immunity, or which immune profiles correlate with long-term protection, allows for the rational selection of vaccine components and adjuvants [10, 12].

### Reduced time-to-development

ML-driven workflows have shortened the time required to progress from antigen discovery to clinical trial readiness. Automated data analysis, high-throughput candidate screening, and in silico trial simulations collectively streamline the vaccine development pipeline, enabling rapid responses to emerging malaria threats [20, 21].

### Enhanced Understanding of immune responses

ML has deepened our understanding of the complex interplay between innate and adaptive immunity in response to malaria vaccination. Analyses of transcriptomic and antibody profiling data have revealed that both the magnitude and quality of immune responses are critical determinants of vaccine efficacy, informing the design of vaccines that induce broad, durable protection [18].

#### Analysis of machine learning applications in malaria vaccine research

Al Meslamani et al. (2024) presented a comprehensive work on the current state of ML applications in infectious diseases that, at the same time, point out the early detection, outbreak, and targeted treatment as potential key areas that can benefit from machine learning [27]. Malik & Waheed (2024) are among those who discuss recent developments in malaria vaccines, mentioning, but do not directly reference, the use of computational methods in enhancing the speed of vaccine development [28]. For further understanding of this perspective, Melchane et al. (2024) provide a comprehensive overview of artificial intelligence for infectious disease prediction and prevention. These general reviews focus on the possible early signs for prediction and individualized therapy [25]. In the case described by Galactionova et al. (2021), the modeling is used in a specific, targeted manner in malaria vaccine policy regarding the implementation of the RTS, S. In particular, the study focuses on a review of several papers that apply mathematical modeling integrated with ML for the purpose of assessing the performance and impact of the RTS S vaccine based on various coverage and cost assumptions. This illustrates how ML can be incorporated into the decision-making system so as to enhance utility gain in immunization activities [23, 24]. Another research by Wistuba-Hamprecht et al. (2024) is focused on identifying the vaccine’s effectiveness level concerning the antibody pattern. Such findings highlight the role of ML in analyzing extensive data to facilitate reasonable vaccine development and assessment [10]. Cross-species data integration notably improved predictive confidence for several antigens, demonstrating the benefit of leveraging evolutionary conservation between *P. falciparum* and *P. vivax* in machine learning-based vaccine discovery frameworks.

### Challenges and future directions: integrating machine learning with emerging technologies

Evidently, the application of ML has brought significant changes in the research on malaria vaccines; however, there are some concerns. Validity and generalizability of the training data, explainability of complex ML models, and combination of outcomes across numerous datasets are some of the issues that persist. However, malaria parasites are biologically diverse, and the immune systems of humans are diversely different, which requires the development of new models that are more general and more reliable.

In the future, the integration of ML with other current developments in the field, like single-cell omics, synthetic biology, and advanced systems immunology, will help to improve the efficiency and effectiveness of vaccine creation [21–26]. Therefore, the progress in malaria vaccine development is further strengthened by the combination of real-time clinical trials and field and laboratory tests.

Al Meslamani et al. (2024) [27] and Melchane et al. (2024) [25] pointed out the limitations of the ML approach, such as the requirement of good data, overfitting, and complications of the large-scale model. Sesay et al. [29] also indicated these challenges include data quality issues, the model’s inherent complexity, and the severe need for validation. The potential for the bankruptcy of healthcare options for citizens must also be considered when it comes to the ethical regulation of the use of AI technologies in healthcare. These challenges call for more research and innovation to enhance the approaches of ML algorithms, the quality and availability of the data, as well as cooperation of computer scientists, immunologists, and personnel in the field of public health. By solving these challenges and advancing ML use, researchers can develop newer and more efficient vaccines against the disease [25, 27, 29].

## Conclusion

Machine learning has influenced malaria vaccine research with possibilities to quickly identify the targets for antigens, determine the effectiveness of the immune response, and speed up the processes of vaccine creation. The role of machine learning in the identification and modelling of effective vaccines defines the value of vaccine scientists, reducing the time taken in the vaccine development process and enhancing understanding of malaria immunology for human health benefit. Integrating machine learning with modern biological technologies in the field will open up new ways of creating highly effective next-generation malaria vaccines that will bring this disease’s eradication closer to reality.

## Supplementary Information

Below is the link to the electronic supplementary material.

Supplementary Material 1

## Data Availability

No datasets were generated or analysed during the current study.
