# Supplementary material for: From data to immunity: the role of machine learning in advancing malaria vaccine research: a scoping review
Source: Trop Dis Travel Med Vaccines. 2025 Oct 28;11:38. doi: 10.1186/s40794-025-00271-2 (PMC12570413; doi:10.1186/s40794-025-00271-2)
Supplement: Supplementary file 1 — Supplementary Material 1 [file 40794_2025_271_MOESM1_ESM.docx]

| **Section and Topic** | **Item #** | **Checklist item** | **Location where item is reported** |
| --- | --- | --- | --- |
| **TITLE** | | |  |
| Title | 1 | Identify the report as a systematic review. | The title clearly identifies the nature of the study as a systematic review: 'From Data to Immunity: The Role of Machine Learning in Advancing Malaria Vaccine Research'. This explicitly signals the review methodology and its focus area, conforming with PRISMA requirements for transparent reporting. |
| **ABSTRACT** | | |  |
| Abstract | 2 | See the PRISMA 2020 for Abstracts checklist. | The abstract is structured and includes Background, Aim, Methods, Results, and Conclusion. It outlines the motivation for the review, the targeted search strategy (focusing on ML in malaria vaccine research), thematic findings across five domains, and a conclusion emphasizing the transformative role of ML. |
| **INTRODUCTION** | | |  |
| Rationale | 3 | Describe the rationale for the review in the context of existing knowledge. | The introduction details the persistent burden of malaria (over 249 million cases in 2022), complexities in Plasmodium falciparum biology, and the limitations of conventional vaccine development. It provides a rationale for the systematic review by introducing the role of machine learning in enhancing research efficiency and accuracy. |
| Objectives | 4 | Provide an explicit statement of the objective(s) or question(s) the review addresses. | The objective is presented at the end of the introduction and reiterated in the abstract: to synthesize and critically evaluate recent applications of machine learning in malaria vaccine research, including antigen discovery, immune profiling, and efficacy prediction. |
| **METHODS** | | |  |
| Eligibility criteria | 5 | Specify the inclusion and exclusion criteria for the review and how studies were grouped for the syntheses. |  |
| Information sources | 6 | Specify all databases, registers, websites, organisations, reference lists and other sources searched or consulted to identify studies. Specify the date when each source was last searched or consulted. | In 'Literature Search and Selection Criteria', the review specifies that a targeted search was conducted for peer-reviewed literature from 2017–2025. Sources included databases and journals with relevance to ML and malaria vaccine research. The search strategy focused on key terms like 'machine learning', 'malaria vaccine', and 'immune profiling'. |
| Search strategy | 7 | Present the full search strategies for all databases, registers and websites, including any filters and limits used. | The search strategy is outlined in the Methods section. Articles were included if they discussed the application of ML or AI in malaria vaccine development. Filters were applied to exclude irrelevant studies such as those with only statistical approaches or lacking a vaccine-related focus. |
| Selection process | 8 | Specify the methods used to decide whether a study met the inclusion criteria of the review, including how many reviewers screened each record and each report retrieved, whether they worked independently, and if applicable, details of automation tools used in the process. | The selection process included screening articles for relevance to ML and malaria vaccines. Only studies meeting predefined inclusion criteria were analyzed. Although the number of reviewers isn’t specified, studies were screened based on title, abstract, and full text to ensure relevance. |
| Data collection process | 9 | Specify the methods used to collect data from reports, including how many reviewers collected data from each report, whether they worked independently, any processes for obtaining or confirming data from study investigators, and if applicable, details of automation tools used in the process. | Data were extracted on ML methods used, data types analyzed, validation techniques, and study outcomes. This included antibody profiles, transcriptomic data, and computational frameworks. Each study was categorized by its thematic focus (e.g., antigen discovery, efficacy modeling). |
| Data items | 10a | List and define all outcomes for which data were sought. Specify whether all results that were compatible with each outcome domain in each study were sought (e.g. for all measures, time points, analyses), and if not, the methods used to decide which results to collect. | Outcomes included antigen identification, predictive modeling of vaccine efficacy, immune signature profiling, and applications of ML in clinical and policy modeling. These outcomes were consistently sought across studies using thematic coding. |
|  | 10b | List and define all other variables for which data were sought (e.g. participant and intervention characteristics, funding sources). Describe any assumptions made about any missing or unclear information. | Additional data items included ML methods (e.g., random forest, support vector machines), data types (e.g., omics, clinical trial results), geographic origin of studies, and validation strategies. Where unclear, assumptions were made based on the publication’s stated methods. |
| Study risk of bias assessment | 11 | Specify the methods used to assess risk of bias in the included studies, including details of the tool(s) used, how many reviewers assessed each study and whether they worked independently, and if applicable, details of automation tools used in the process. | Quality assessment involved evaluating methodological rigor such as the ML model’s appropriateness, robustness of validation (e.g., cross-validation), and reporting clarity. Studies using innovative techniques like positive-unlabeled learning were critically appraised for validity. |
| Effect measures | 12 | Specify for each outcome the effect measure(s) (e.g. risk ratio, mean difference) used in the synthesis or presentation of results. | Effect measures varied: for antigen prediction, model performance metrics such as accuracy, ROC-AUC, and cross-validation scores were reported; for immune profiling, effect sizes were expressed as classification accuracy and predictive robustness. |
| Synthesis methods | 13a | Describe the processes used to decide which studies were eligible for each synthesis (e.g. tabulating the study intervention characteristics and comparing against the planned groups for each synthesis (item #5)). | Studies were grouped into five thematic areas: (1) Antigen discovery, (2) Immune profiling, (3) Tool development, (4) Review articles, (5) Epidemiological modeling. These groupings facilitated synthesis and comparison. |
|  | 13b | Describe any methods required to prepare the data for presentation or synthesis, such as handling of missing summary statistics, or data conversions. | Data were prepared through thematic classification, with incomplete or missing data acknowledged in the Discussion. Some studies lacked detailed validation metrics, and this was noted during synthesis. |
|  | 13c | Describe any methods used to tabulate or visually display results of individual studies and syntheses. | Results were displayed in structured summaries using concept maps, heatmaps, and thematic tables. For example, a heatmap showed confidence scores for antigen prediction using various ML techniques. |
|  | 13d | Describe any methods used to synthesize results and provide a rationale for the choice(s). If meta-analysis was performed, describe the model(s), method(s) to identify the presence and extent of statistical heterogeneity, and software package(s) used. | Narrative synthesis was used due to heterogeneity in study types. Where applicable, results were summarized by study focus and ML methodology, without statistical pooling. |
|  | 13e | Describe any methods used to explore possible causes of heterogeneity among study results (e.g. subgroup analysis, meta-regression). | Sources of heterogeneity included variability in ML models, data types (e.g., genomic vs. clinical), study designs, and regional data representation. These were discussed in context in the Discussion. |
|  | 13f | Describe any sensitivity analyses conducted to assess robustness of the synthesized results. | No formal sensitivity analyses were performed. The review acknowledges this as a limitation due to the nature of the included studies and their qualitative synthesis. |
| Reporting bias assessment | 14 | Describe any methods used to assess risk of bias due to missing results in a synthesis (arising from reporting biases). | Risk of bias due to missing results was considered in the Discussion. The authors acknowledge potential publication bias and underrepresentation of studies from low-income regions. |
| Certainty assessment | 15 | Describe any methods used to assess certainty (or confidence) in the body of evidence for an outcome. | Certainty was discussed through analysis of study quality, validation robustness, and consistency of findings across themes. Higher confidence was placed in studies with rigorous ML validation and transparent data. |
| **RESULTS** | | |  |
| Study selection | 16a | Describe the results of the search and selection process, from the number of records identified in the search to the number of studies included in the review, ideally using a flow diagram. |  |
|  | 16b | Cite studies that might appear to meet the inclusion criteria, but which were excluded, and explain why they were excluded. |  |
| Study characteristics | 17 | Cite each included study and present its characteristics. | Characteristics such as study region (e.g., Europe, North America, Africa), ML methods used, and vaccine development stage addressed were summarized in the Results section under thematic categories. |
| Risk of bias in studies | 18 | Present assessments of risk of bias for each included study. | Risk of bias in individual studies was qualitatively assessed based on model selection, data quality, and whether validation was internal or external. Limitations were discussed for studies lacking robust validation. |
| Results of individual studies | 19 | For all outcomes, present, for each study: (a) summary statistics for each group (where appropriate) and (b) an effect estimate and its precision (e.g. confidence/credible interval), ideally using structured tables or plots. | Each study’s key findings are described, including ML techniques used and predictive outcomes (e.g., immune signatures, antigen ranking). Performance metrics like classification accuracy and ROC-AUC were reported. |
| Results of syntheses | 20a | For each synthesis, briefly summarise the characteristics and risk of bias among contributing studies. | Each thematic synthesis summarizes contributing studies, their ML methods, risk of bias, and study limitations. For example, studies with incomplete antigen data were flagged as having moderate bias. |
|  | 20b | Present results of all statistical syntheses conducted. If meta-analysis was done, present for each the summary estimate and its precision (e.g. confidence/credible interval) and measures of statistical heterogeneity. If comparing groups, describe the direction of the effect. | No statistical meta-analysis was performed; instead, findings were synthesized narratively by thematic category and methodology. |
|  | 20c | Present results of all investigations of possible causes of heterogeneity among study results. | Causes of heterogeneity discussed include differences in ML algorithms, sample sizes, input data types, and validation standards. These impact generalizability. |
|  | 20d | Present results of all sensitivity analyses conducted to assess the robustness of the synthesized results. | Sensitivity analyses were not performed. This was acknowledged as a limitation due to heterogeneity and lack of comparable effect sizes. |
| Reporting biases | 21 | Present assessments of risk of bias due to missing results (arising from reporting biases) for each synthesis assessed. | The risk of reporting bias was addressed in the Discussion. The authors note potential overrepresentation of studies with positive outcomes and lack of access to unpublished data. |
| Certainty of evidence | 22 | Present assessments of certainty (or confidence) in the body of evidence for each outcome assessed. | Certainty of evidence was qualitatively discussed, with greater confidence in themes supported by multiple high-quality, independently validated studies. Limitations were highlighted for single-study findings. |
| **DISCUSSION** | | |  |
| Discussion | 23a | Provide a general interpretation of the results in the context of other evidence. |  |
|  | 23b | Discuss any limitations of the evidence included in the review. |  |
|  | 23c | Discuss any limitations of the review processes used. |  |
|  | 23d | Discuss implications of the results for practice, policy, and future research. |  |
| **OTHER INFORMATION** | | |  |
| Registration and protocol | 24a | Provide registration information for the review, including register name and registration number, or state that the review was not registered. | No formal registration of the review was undertaken. |
|  | 24b | Indicate where the review protocol can be accessed, or state that a protocol was not prepared. | No review protocol was developed prior to the conduct of the review. |
|  | 24c | Describe and explain any amendments to information provided at registration or in the protocol. | As no protocol was registered, no amendments were applicable or required. |
| Support | 25 | Describe sources of financial or non-financial support for the review, and the role of the funders or sponsors in the review. | Acknowledgment section explicitly states that the review was supported by Dubai Medical College for Girls. No role of funders in the review process was reported. |
| Competing interests | 26 | Declare any competing interests of review authors. | Authors declared that there were no competing interests influencing the review. |
| Availability of data, code and other materials | 27 | Report which of the following are publicly available and where they can be found: template data collection forms; data extracted from included studies; data used for all analyses; analytic code; any other materials used in the review. | All data and materials (including thematic classifications and figures) are included within the manuscript. No additional data repositories were used. |

*From:*  Page MJ, McKenzie JE, Bossuyt PM, Boutron I, Hoffmann TC, Mulrow CD, et al. The PRISMA 2020 statement: an updated guideline for reporting systematic reviews. BMJ 2021;372:n71. doi: 10.1136/bmj.n71. This work is licensed under CC BY 4.0. To view a copy of this license, visit <https://creativecommons.org/licenses/by/4.0/>
